# Impact of Gas Flow and Humidity on Trans-Nasal Aerosol Deposition via Nasal Cannula in Adults: A Randomized Cross-Over Study

**DOI:** 10.3390/pharmaceutics11070320

**Published:** 2019-07-07

**Authors:** Luciana Alcoforado, Arzu Ari, Jacqueline de Melo Barcelar, Simone Cristina S. Brandão, James B. Fink, Armele Dornelas de Andrade

**Affiliations:** 1 Department of Physical Therapy, Universidade Federal de Pernambuco, Recife 50740-560, PE, Brazil; 2 Department of Respiratory Therapy, Texas State University, Round Rock, TX 78665, USA; 3 Medicine Nuclear Department, Universidade Federal de Pernambuco, Recife 50670-901, PE, Brazil; 4 Aerogen Pharma Corp, San Mateo, CA 94402, USA; 5 Avenida Jornalista Aníbal Fernandes, SN—Cidade Universitária, CEP, Recife 50740-560, PE, Brazil

**Keywords:** nasal cannula, humidity, aerosol, scintigraphy, oxygen and nebulizer

## Abstract

Background: Trans-nasal pulmonary aerosol delivery using high flow nasal cannula (HFNC) devices is described with the administration of high gas flows exceeding patient inspiratory flow (HF) and with lower flows (LF). The aim of this pilot clinical trial was to compare deposition and distribution of radiolabeled aerosol via nasal cannula in healthy adults across three rates of gas flow delivered with active heated humidification, and to further identify the impact of aerosol administration without heated humidity. Methods: Twenty-three (23) healthy adults (16F) were randomized to receive aerosol with active heated humidification or unheated oxygen at gas flows of 10 L/min (*n* = 8), 30 L/min (*n* = 7), or 50 L/min (*n* = 8). Diethylenetriaminepentaacetic acid labeled with 1 millicurie (37 MBq) of Technetium-99m (DTPA-Tc99m) was mixed with NaCl to a fill volume of 1 mL, and administered via mesh nebulizer placed at the inlet of the humidifier. Radioactivity counts were performed using a gamma camera and the regions of interest (ROIs) were delimited with counts from the lungs, upper airways, stomach, nebulizer, circuit, and expiratory filter. A mass balance was calculated and each compartment was expressed as a percentage of the total. Results: Lung deposition (mean ± SD) with heated humidified gas was greater at 10 L/min than 30 L/min or 50 L/min (17.2 ± 6.8%, 5.71 ± 2.04%, and 3.46 ± 1.24%, respectively; *p* = 0.0001). Using unheated carrier gas, a lung dose of aerosol was similar to the active heated humidification condition at 10 L/min, but greater at 30 and 50 L/min (*p* = 0.011). Administered gas flow and lung deposition were negatively correlated (*r* = −0.880, *p* < 0.001). Conclusions: Both flow and active heated humidity inversely impact aerosol delivery through HFNC. Nevertheless, aerosol administration across the range of commonly used flows can provide measurable levels of lung deposition in healthy adult subjects (NCT 02519465).

## 1. Introduction

Oxygen administration via high flow nasal cannula (HFNC) supports acute and critically ill patients with respiratory failure [1,2,3,4,5]. HFNC therapy promotes oxygenation, generation of positive airway pressure, reduced rebreathing of carbon dioxide, and increased comfort when compared to other methods [4,5,6,7,8,9]. Patients receiving HFNC may benefit from inhaled medications. Trans-nasal pulmonary administration of medical aerosols using HFNC devices has been reported with gas flow exceeding patient inspiratory demand (high flow) and gas delivery lower than patient inspiratory flow (LF) [10]. 

Administration of oxygen via HFNC presents a variety of challenges for efficient aerosol delivery. High gas flow dilutes aerosol, and generates transitional and turbulent flows in narrow circuits and cannula, thus increasing impacting losses of aerosol and reducing the amount of aerosol available to be inhaled. Additionally, the nasopharynx filters aerosols, thereby increasing deposition in the upper airways and reducing the therapeutic dose in the lungs [5,9,11,12].

In vitro studies have reported a reduction of inhaled dose as system delivery flow increases [6,13,14] and the diameter of the nasal cannula decreases [15]. Aerosol deposition reported with different systems have varied by an order of magnitude based on the type of humidifier, nebulizer, adapter, and placement used.

In vitro testing of aerosol delivery during mechanical ventilation commonly report inhaled dosage with unheated versus heated humidified gas, favoring unheated delivery by >60% [16]. More recently, the use of models simulating active heated humidified exhalation have reported smaller differences of the inhaled dose between unheated and heated humidified gas than models which do not heat and humidify exhaled gas. The implication is that models without active heated exhalation may overestimate the inhaled dose with unheated gas compared to in vivo delivery [17]. Although clinical administration of anhydrous unheated oxygen at flows greater than 6 L/min (American College of Chest Physicians) is not recommended, we aimed to assess the aerosol delivery with heated humidified and unheated gas in vivo to better understand the relevance of those in vitro observations.

In vitro studies [6,9,13,14,15] differ as to whether aerosol administered via HFNC can provide clinically relevant therapeutic levels of aerosols to the lungs; however, an in vivo study of radiolabeled aerosol by Dugernier et al. [18] observed low deposition (3.6%) of aerosol from a mesh nebulizer via high-flow nasal cannula at a single flow of 30 L/min; however, it remains unknown if the higher deposition observed with lower flow rates on the bench translates to greater lung delivery in humans. 

Thus, the behavior of trans-nasal pulmonary deposition of aerosol at both lower and elevated flows, as well as the influence of unheated vs. active heated humidification using HFNC devices in humans is not yet known [4,5]. Clinical studies to quantify pulmonary deposition of aerosol via HFNC devices are necessary to provide guidance on the use of administrating high and low gas flows in clinical practice.

Our hypothesis was that trans-nasal pulmonary aerosol delivery using a HFNC device:

(1) can deliver measurable quantities of aerosol to the lungs;
(2) has greater delivery efficiency with lower system gas flow; and
(3) varies when administered with active heated humidification rather than unheated gas.

Therefore, the aim of this study was to compare the effect of gas flow and active heated humidification on the deposition and distribution of radiolabeled aerosol from a vibrating mesh nebulizer (VMN) during administration via HFNC setup in healthy adult subjects.

## 2. Methods 

### 2.1. In Vivo Study Design and Sample 

A randomized, cross-over pilot clinical study of healthy volunteers was performed at the Nuclear Medicine Department of the Hospital das Clínicas/Universidade Federal de Pernambuco in Recife, Brazil, and was approved by the Research and Ethics Committee on Humans (no. 54705616700005208 with Clinical Trials Registry (no. NCT 02519465). Informed consent was obtained from all individual participants included in the study.

**Figure 1:**
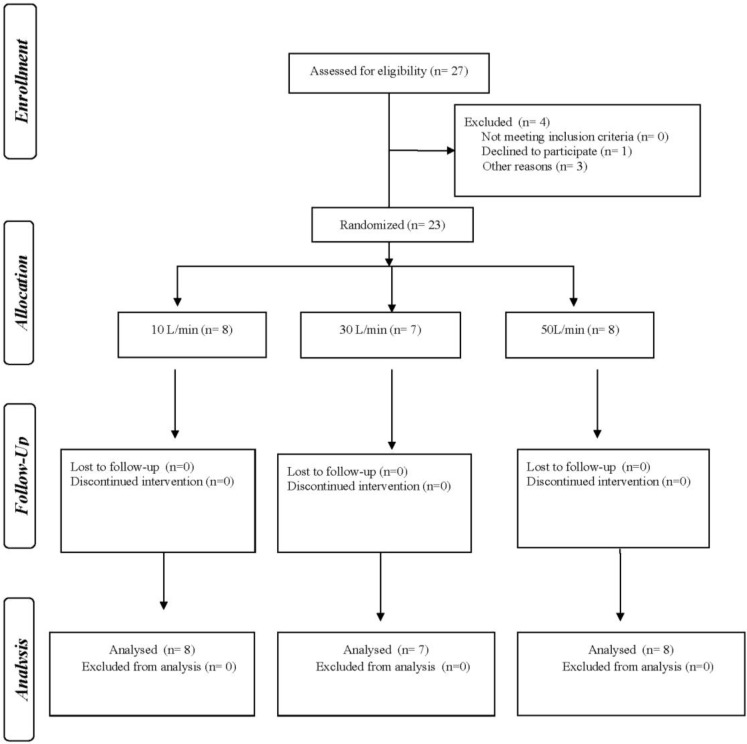
Study flow chart.

Consenting volunteers were randomly allocated to receive radiolabeled aerosol via HFNC with heated humidified and unheated gas (crossover) at gas flows of 10, 30 or 50 L/min, with ≥7-day washout between administrations (Figure 1). 

Two researchers were involved. The first generated random tables (http://www.randomization.com) and managed sealed envelopes, while the second administered inhalation and image acquisition. Subjects were blinded to administered flow and active heated humidification. 

Healthy volunteers of both genders between 18–65 years, without a history of lung disease, with forced vital capacity (FVC) or forced expiratory volume in the first second (FEV1) ≥ 80% of predicted values [19] were included. Exclusion criteria were a history of smoking, diagnosed lung disease, active rhinitis, sinusitis, or pregnant women.

### 2.2. Procedures and Measurements

#### 2.2.1. Initial Clinical Evaluation

The initial evaluation included age, gender, body mass index (BMI), respiratory rate (RR), blood pressure (AP0316, CBEMED, BIC, São Paulo, Brazil) with oxygen saturation (SpO_2_) and heart rate (HR) (pulse oximeter, Onyx^®^ Vantage 9590, Plymouth, MN, USA). Spirometry (Micro Loop 8/Cardinal Health, England, UK) followed the American Thoracic Society [20] guidelines. 

#### 2.2.2. Aerosol Administration 

**Figure 2:**
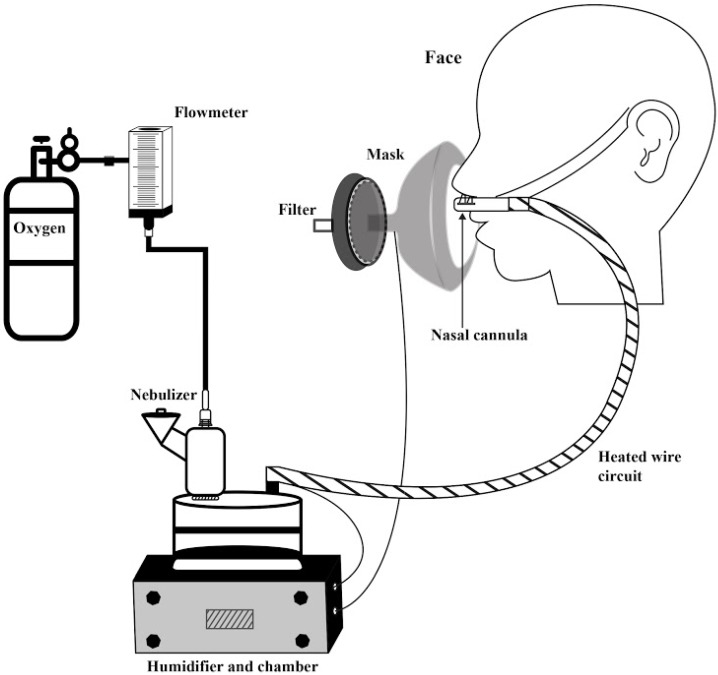
High flow nasal cannula system consisting of compressed oxygen cylinder with regulator and pressure compensated flowmeter, connecting to a T-piece with vibrating mesh nebulizer attached to the inlet of the humidifier chamber, with the outlet connected to a heated wire circuit attached to a nasal cannula and placed in the nares of the subject. A mask with a collecting filter was placed over the face and cannula to collect exhaled and escaped aerosol.

Diethylenetriaminepentaacetic acid labeled with 1 millicurie (37 MBq) of Technetium-99m (DTPA-Tc99m) in 0.9% saline to a total volume of 1 mL was administered via vibrating mesh nebulizer (VMN: Aerogen Solo, Aerogen Ltd., Galway, Ireland) placed at the inlet of a passover humidifier filled with sterile water and attached to a corrugated heated wire tubing and medium-sized adult nasal cannula (Optiflow^TM^; Fisher&Paykel Healthcare, Auckland, New Zealand) (Figure 2).

Oxygen was dispensed from a calibrated back pressure compensated flowmeter at 10, 30, and 50 L/min. Gas passed through the nebulizer connector, carrying aerosol into the inlet of the humidifier filled with water. For heated humidity, the water in the humidifier and circuit were heated to 34–36 °C. For unheated conditions, water in the humidifier was room temperature (20–22 °C) and the circuit was unheated.

After device setup and temperature stabilization, subjects were seated and nasal cannula prongs were placed in the nostrils. An orofacial mask with filter (Vital Signs, San Diego, California, USA) was placed over the cannula and lightly sealed to the face. Subjects were instructed to breathe normally and allotted 2 min to acclimate to the setup prior to dosing. The 1 mL dose was placed in the nebulizer reservoir and administered to completion.

#### 2.2.3. Lung Scintigraphy

To sample the posterior thorax, subjects were seated close to the gamma camera detector (Starcam 3200 AC/T GE Medical Systems, Little Chalfont, Buckinghamshire, UK) with an acquisition of 300 s with a matrix of 256 × 256 pixel. The scanner was repositioned to scan the anterior upper airway/face, followed by a scan of device components (nebulizer, humidifier chamber, tubing, cannula, mask, and filter) [21].

Both pulmonary and extrapulmonary regions of interest (ROI) were delimited using the Xeleris 3 Functional Imaging Workstation (GE Healthcare, Milwaukee, WI, USA). The radiation count of each compartment (lungs, upper respiratory tract, stomach, device, and filter) was determined for each ROI, with a mass balance expressed as a percentage of the sum of the counts (primary outcomes) [22]. Attenuation and tissue absorption correction factors for lungs, stomach, and oropharynx were applied as described by Lee [23]. Correction factors of 2.27 were specifically applied to the lung and stomach counts, and 2.37 for the upper airway, with no correction applied to device components and filters. 

#### 2.2.4. Particle Size Characterization

The mass median aerodynamic diameter (MMAD) and geometric standard deviation (GSD) of aerosol exiting the HFNC under conditions described above were determined by a cascade impactor (Andersen Cascade Impactor; Thermo, Atlanta, GA, USA) operated at 28.3 L/min. Radiolabeled aerosol with gas rates of 10, 30, and 50 L/min was sampled for 2 min with throat and impactor stages scanned using 2D scintigraphy for 300 s, with each stage counted as ROI. Counts were used to calculate MMAD and GSD with software (http://www.mmadcalculator.com).

### 2.3. Statistical Analysis

The study sample size was calculated after a pilot study with five volunteers in each group (10, 30, and 50 L/min) using (http://hedwig.mgh.harvard.edu/sample_size/size.html). The sample size calculation was made as a superiority study with primary endpoint of pulmonary deposition at flows of 10 L/min (49,522 ± 18,693), 30 L/min (42,953 ± 21,529), and 50 L/min (32,872 ± 12,227). A total of 15 individuals with allocation of 5 volunteers in each group was estimated based on the alpha level and power set to 0.05 and 80%, respectively.

Sample distribution was analyzed using the Shapiro–Wilk and Levene tests. The non-categorical variable was evaluated with Fisher’s exact test. We used the one-way analysis of variance (ANOVA) to compare flow rates with the Tukey post-hoc test for parametric variables and the Kruskal–Wallis test for non-parametric variables. Comparisons in aerosol deposition between heated humidified and unheated systems were performed using the paired sample *t*-test for parametric variables and Mann–Whitney U test for non-parametric variables. The Pearson and Spearman correlation were used to assess correlation between variables. Data were processed with SPSS 18.0 statistical software (SPSS Inc., Chicago, IL, USA) (*p* < 0.05). Aerosol deposition was expressed as percentage (mean ± SD%) of nominal dose placed in the nebulizer [22].

## 3. Results

Of the 27 subjects screened, 23 participated in the study with 8 allocated to receive a flow of 10 L/min, 7 of 30 L/min, and 8 of 50 L/min. (Figure 1). Anthropometric characteristics and spirometric measures were similar for all three groups (Table 1). The dosing time ranged from 2–4 min. 

### 3.1. Lung Deposition

Table 2 shows percentage of aerosol deposition (mean ± SD) during HFNC with active heated humidification. 

**Figure 3:**
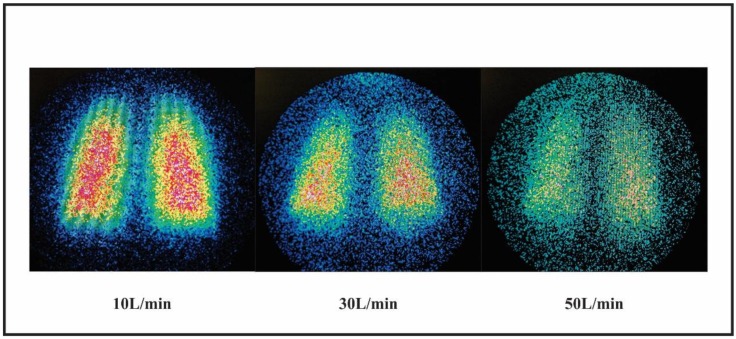
Representative images of pulmonary deposition with heated humidified LFNC at 10 L/min and HFNC at 30 L/min and 50 L/min.

A negative correlation of lung deposition and flow rate was observed with and without active heated humidification (*r* = −0.874/*p* < 0.001 and *r* = −0.572/*p* < 0.001, respectively). Representative scintigraphy images of pulmonary deposition with active heated humidified condition at 10, 30, and 50 L/min are shown in Figure 3.

**Figure 4:**
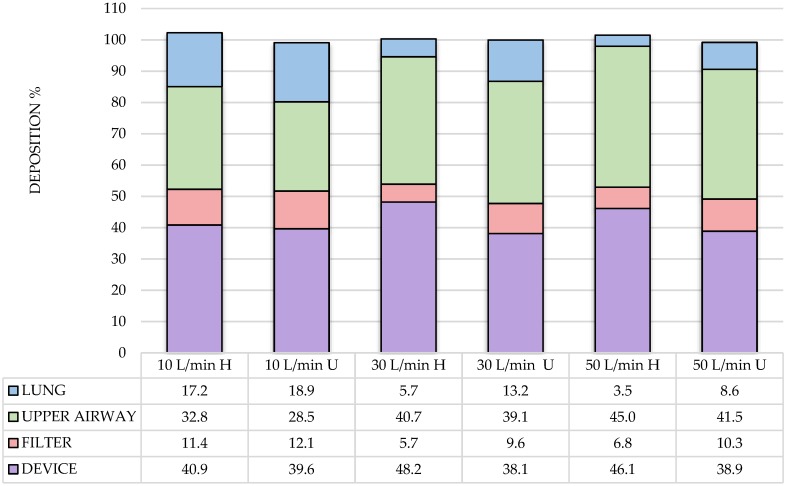
Aerosol distribution across compartments with LF and HFNC during heated humidified (H) and unheated (U) at flows of 10, 30, and 50 L/min.

Lung delivery of aerosol with unheated gas via HFNC was greater than with active heated humidification at 30 L/min (13.16 ± 6.78% vs. 5.71 ± 2.04%, respectively; *p* = 0.015) and 50 L/min (8.59 ± 2.54% vs. 3.46 ± 1.24%, *p* < 0.001). However, at 10 L/min lung deposition with active heated humidification (17.23 ± 6.78%) and without (18.86 ± 10.01) were similar (*p* = 0.531) (Figure 4). 

#### 3.1.1. Aerosol Deposition in the Device

A positive correlation was observed between deposition in the nasal cannula and flow with (*r* = 0.778, *p* < 0.001) and without active heated humidification (*r* = 0.597, *p* < 0.001) conditions, respectively. There was a negative correlation between the amount of drug deposited in the nasal cannula and lung dose with active heated humidification using HFNC (*r* = −0.665, *p* < 0.001), and with unheated gas (*r* = −0.606, *p* < 0.01). 

#### 3.1.2. Aerosol Deposition in the Expiratory Filter and Mask

Expiratory filter deposition was lower at 30 and 50 than 10 L/min (*p* = 0.014) (Table 2). A negative correlation was found between flow rate and drug deposition in the expiratory filter with active heated humidification conditions (*r* = −0.456, *p* = 0.029). 

### 3.2. MMAD Results

MMAD of aerosol exiting the cannula at 10, 30, and 50 L/min was greater with active heated humidification than unheated gas (2.29 ± 0.22 μm vs. 1.29 ± 0.22 μm; *p* = 0.038) with no trend in particle size change across flows. In contrast, GSD with heated humidification was similar to unheated gas (1.45 ± 0.18 vs. 1.6 ± 0.25, respectively), independent of flow.

## 4. Discussion 

To our knowledge, this is the first study to quantify pulmonary delivery of radiolabeled aerosol administered via HFNC to adult subjects across a range of flows, confirming that flow and heated humidity impact lung dose. 

Our lung dose at 30 L/min with active heated humidity (5.71 ± 2.04) is consistent with that reported by Dugernier et al. [18], who observed a lung deposition of 3.6% with a 4 mL (4 miC) fill volume using the same model of VMN at 30 L/min with heated humidity, as determined by single photon emission computed tomography (SPECT). It should be noted that the difference in fill volume used (1 and 4 mL) was not associated with differences in lung delivery efficiency. They reported 2.6% dose retention in the VMN compared to our 6%. The retention in the jet nebulizer which they used was 45.0%, which could partly account for the lower deposition in comparison with VMN. The authors [18] estimated loss of exhaled and escaped aerosols trickling from the nose at 20.5% in contrast to our measured losses of 4.3% in the expiratory filter and mask. In contrast, we report an upper airway deposition (42.1%) which was similar to their combined upper airway and nasopharyngeal compartments of 35.5%. 

### 4.1. Influence of Flow Rate on Pulmonary Deposition

The inverse correlation of administered gas flow to inhaled lung dose are consistent with in vitro reports. Using a casting of an adult airway, Reminiac et al. [6] reported reductions of aerosol delivery efficiency distal to the trachea with flows of 30, 45, and 60 L/min (6.7%, 3.5%, and 3%, respectively). Measuring the dose distal to the cannula at flows of 10, 30, and 50 L/min, Dailey et al. [17] reported inhaled dose efficiency of 26.7%, 11.6%, and 3.5%, respectively. Differences between the two models may be due to the collection point, with higher delivery efficiency in the simulated nose than distal to the trachea.

In contrast and using the same VMN with a different humidifier system (Vapotherm), adapter and nebulizer position, Perry et al. [14] reported lower deposition by >10 fold than other models at flows of 5 (2.5%), 10 (0.8%), 20 (0.4%), and 40 L/min (0.2%), respectively. The authors concluded that the low delivery efficiency of aerosol with HFNC would not be suitable for effective therapeutic drug delivery to adults. Our findings are more consistent with the in vitro reports of Ari, Dailey, and Reminiac, and suggest that a measurable and potentially therapeutic lung dose can be achieved with HFNC. 

### 4.2. Particle Size Distribution

The particle size distribution of aerosol exiting the VMN was measured as 3.9 µm MMAD with 2.1 GSD. We found that aerosol exiting the cannula during HFNC was larger with active heated humidity as carrier gas, with no emitted particles greater than 2.6 µm. We observed a trend to larger aerosol particle size distribution with active heated humidified versus unheated gas; however, the difference in aerosol MMAD exiting the cannula in both conditions was not flow dependent, and likely not of clinical significance. This suggests that much of the impacted loss of generated aerosol occurs en route through the circuit before reaching the cannula. 

Bhashyam et al. [15] reported volume median diameter of aerosol particles emitted by the VMN to be 5 µm, with a reduction to 1.9–2.2 µm exiting the cannula at 3 L/min, with variations dependent on the size of cannula. Reminiac et al. [6] reported a MMAD of 1.8 μm with a GSD of 1.9. In contrast, Perry et al. [14] reported MMAD of 0.61 µm with GSD of 9.6 at 10 L/min, and 4.8 µm with GSD of 9.5 at 40 L/min. The greater variability in range of MMAD across the two flows and the higher GSDs could be attributed to the adapter used and placement with the implemented Vapotherm HFNC system, as well as condensation droplets accumulating and spraying from the cannula outlet. 

A VMN producing 3.9 µm particles at the inlet of the humidifier results in larger particles impacting in the humidifier and connecting tubing prior to entering the cannula, thereby reducing the volume of particles impacting in the cannula. This reduces liquid incidence building up in the cannula and the frequency of spraying larger droplets into the nose. 

The decrease in inhaled aerosol with increasing flows during HFNC is related to two factors: (1) increased transitional flows and turbulence promoting greater inertial particle impaction within the device and airways, reducing the mass of aerosol available for inhalation; and (2) dilution of aerosol as gas flows exceed the inspiratory flow of the subject, reducing the concentration of inhaled aerosol, decreasing the inhaled aerosol mass/L.

### 4.3. Heating and Humidifying Influence Pulmonary Deposition

Heated humidification is commonly used during nasal oxygen greater than low flows of 4–6 L/min [24]. High flow rates of anhydrous gas can cause dryness in the nose, mouth, and throat and irritate mucosa, increasing nasal resistance and bronchial hyper-responsiveness. Consequently, providing heat and humidity is considered essential, even at the risk of reduced pulmonary aerosol delivery [25,26,27]. The use of active heated humidity with HFNC has been associated with greater comfort, tolerance, and lower respiratory rate [12,24]. This is consistent with our findings.

Pulmonary deposition of the aerosol with lower flow (10 L/min) was similar in comparing deposition with and without active heated humidified gas. This may be partly due to the function of the upper airway in heating and humidifying gas on inspiration so that change in particle size occurred prior to passing through the lower airways. 

Aerosol administered with active heated humidity during ventilator support is associated with lower aerosol delivery efficiency attributed to hygroscopic particle growth [27] in transit, with subsequent greater impacting losses in circuit components and airways. However, it appears that particle size changes in response to high absolute humidity may occur secondary to the subject’s exhaled humidity, resulting in similar lung deposition efficiency at 10 L/min with and without active heated humidification condition, but not at higher flows. It is possible that the capacity of the nose to heat and humidify inhaled gas is exceeded in the presence of higher flows, and the isothermal saturation boundary (the point at which high absolute humidity is achieved) moves lower in the airways [24]. As administered gas flow increases, the volume passing through the cannula exceeds the ability of the upper airways to provide sufficient absolute humidity, it is likely that the particle size does not increase as much with the unheated condition, resulting in a higher lung dose.

Previous studies [24,25,28,29,30] have reported that the deposition of aerosol using pressurized metered-dose inhalers (pMDI) and nebulizers can be reduced by 50% when delivered gas is heated and humidified versus dry. Miller et al. [31] confirmed reduced delivery with heated humidification via ETT in vivo. Our findings of reduced aerosol delivery at higher flows (30 and 50 L/min) with active heated humidity compared to unheated gas are consistent with Miller et al. [31]. 

### 4.4. Aerosol Therapy and HFNC

We demonstrated pulmonary aerosol delivery ranging from 3.5% to 17.2%. Lower deposition at high flow may be sufficient for administration of drugs like bronchodilators, but not necessarily sufficient for therapeutic dosing with other drug classes. MacIntyre et al. [32] reported lung doses of 2–3% in ventilated subjects, with Fuller et al. [33] reporting even lower deposition from jet nebulizers during mechanical ventilation. Duarte et al. [34] reported bronchodilator response in ventilated patients with jet nebulizers under similar conditions, while Dugernier et al. [18] reported lower lung dose with JN (1%) than VMN (3.6%) with similar flows, albeit the placement of nebulizers were different. Bräunlich and Wirtz [35] compared administration of a standard dose of short acting bronchodilator via jet nebulizer via HFNC at gas flow of 35 L/min compared to nebulizer with mouthpiece, reporting similar pre and post bronchodilator response in both arms. Similarly, in a randomized control trial of 25 subjects with obstructive airways disease, they received 2.5 mg of albuterol via VMN during HFNC at 30 L/min and by jet nebulizer with a facemask. Furthermore, Reminac et al. [36] reported that albuterol vibrating mesh nebulization within a nasal high-flow circuit induced similar bronchodilation to standard facial mask jet nebulization. They concluded that beyond pharmacological bronchodilation, nasal high flow by itself may induce small but significant bronchodilation. 

### 4.5. Clinical Implications

Nasal cannula over any flow range from low to high may be a useful way to deliver aerosols in general, thus representing another tool available to the clinician. Compared to oral delivery, nasal delivery may have some advantages, particularly at higher flow where oral aerosol delivery may interrupt the benefits in oxygenation, CO_2_ reductions, and positive airway pressure associated with HFNC. Administering aerosol via oral routes while the subject receives HFNC greatly reduces the inhaled dose [37]. However, any possible clinical advantages at this stage of development are remain speculative. 

Our findings demonstrate that it is possible to achieve measurable transnasal pulmonary delivery under the conditions tested, and that further work in acutely ill patients is required to demonstrate whether these levels of delivery can achieve desired clinical endpoints for aerosol administration of specific agents to the lung via HFNC. The low flow of 10 L/min provided greater aerosol to the lung (17%) than would be expected with standard jet nebulizer using a mouthpiece or mask (8–12%) [32], representing the range of lung dose associated with the jet nebulizers commonly used in the clinical trials for many of the approved nebulizer formulations for inhalation. For prolonged administration masks (for oxygen or aerosol) tend to be problematic as they are difficult to seal properly, can be uncomfortable and difficult for patient to speak or take nourishment. Standard oxygen masks at 10 L/min typically deliver FIO2 in the range of 30–40%, but only when firmly and securely seated on the face. Not all patients require the higher range of gas flow to support oxygenation. For patients with lower FIO2 requirements, flows of 10 L/min or less are common. Most adults have peak inspiratory flows of 30 L/min and mean inspiratory flows of 14 L/min at rest, but peak inspiratory flow may increase to 45 or 60 L/min when patients are in respiratory distress. For severely hypoxic patients, the higher flows are common to achieve FIO2 of 0.7 or greater, so not all patients on HFNC would be expected to tolerate the lower 10 L/min flow.

## 5. Limitations

This study was conducted in normal healthy adult volunteers with relatively consistent non-stressed respiratory rate, tidal volume, and inspiratory capacity. Our findings may underestimate pulmonary delivery for patients with distressed breathing patterns as shown in vitro [6,18]. In contrast, using a mask with a filter to collect exhaled and fugitive aerosols allowed us to quantify a key compartment comprising the mass balance; however, the mechanical dead space of the mask may have had a reservoir effect, thereby slightly increasing the inhaled upper airway and lung dose. 

There is inherent variability in the emitted dose rate and particle size distribution of the commercial ‘off the shelf’ vibrating mesh nebulizer used in this study, Aerosol output ranges from 0.2–0.5 mL/min resulting in administration times of a 1.0 mL dose ranging from 2–4 min. Our reported particle size distribution of aerosol exiting the VMN of 3.9 μm MMAD is greater than the range of 2.0–3.2 μm reported by the manufacturer (FDA 510(k) K133360). Device variability and the different analytic methods (assay of albuterol sulfate with UV/VIS vs. 2D scintigraphy of radiolabeled aerosol) may explain these differences. Nevertheless, the reduction of aerosol MMAD exiting the cannula is consistent with other reports [6,15].

We only reported placement of the VMN at the inlet of the humidifier, consequently HFNC with nebulizer placed elsewhere in the circuit may change inhaled delivery efficiency. Placement of aerosol devices between humidifier and patient results in greater rainout of aerosol particles in the tubing with more frequent occlusion of the nasal prongs.

Deposition results for radiolabeled aerosol delivery are very sensitive to attenuation correction and it has been well documented that attenuation corrections vary between individuals. Due to the limitation of available technology on site, we did not measure these effects for each subject, but rather applied a ‘representative’ factor across subjects. These representative values may result in potential errors compared to individual values. In addition, the systemic and clinical consequences of relatively high nasopharyngeal deposition of aerosol of specific medications were not addressed in this study. Future clinical work should assess the side effects of associated systemic exposure with specific inhaled medications.

## 6. Conclusions

Both flow and active heated humidity inversely impact aerosol delivery through HFNC. Nevertheless, aerosol administration across the range of commonly used flows can provide measurable levels of lung deposition in healthy adult subjects. Further studies in acutely ill patients are warranted to evaluate dosing strategies for effective drug delivery via HFNC across flows.

## Figures and Tables

**Table 1:** Anthropometric characteristics and spirometric measures of participants who received inhalation therapy through HFNC at 10 L/min, 30 L/min, and 50 L/min.

Parameter	10 L/min (*n* = 8)	30 L/min (*n* = 7)	50 L/min (*n* = 8)	*p*-Value
AGE (years)	30.88 ± 8.34	24.00 ± 3.05	26.63 ± 5.4	0.115
Gender	2 M/6 F	2 M/5 F	3 M/5 F	0.856
BMI (Kg/m^2^)	22.24 ± 2.25	25.62 ± 1.48	24.64 ± 3.60	0.058
HR (bpm)	78.57 ± 6.21	88.33 ± 5.39	77.00 ± 9.41	0.099
RR (ipm)	13.57 ± 4.07	15.33 ± 2.73	13.83 ± 3.65	0.650
SpO_2_ (%)	98.14 ± 0.69	97.83 ± 1.16	98.14 ± 0.69	0.765
IC (L)	2.27 ± 0.34	2.44 ± 0.45	2.52 ± 0.59	0.646
Tidal Volume (L)	0.91 ± 0.33	0.77 ± 0.15	0.70 ± 0.52	0.599
FEV_1_ (%pred)	94.00 ± 10.11	90.67 ± 8.31	89.00 ± 6.57	0.594
FVC (%pred)	93.33 ± 9.00	89.33 ± 6.77	89.33 ± 6.12	0.570
PEF (%pred)	82.20 ± 18.95	82.40 ± 6.46	81.40 ± 4.61	0.990
FEV_1_/FVC (%pred)	100.0 ± 5.17	100.17 ± 6.85	98.17 ± 7.38	0.843

Data is expressed as mean ± standard deviation. One-way Anova, *p* < 0.05. BMI = body mass index, HR = heart rate, RR = respiratory rate, SpO_2_ = oxygen saturation, IC = inspiratory capacity, FEV_1_ (%pred) = percentage of predicted for forced expiratory volume in 1 s, FVC (%pred) = percentage of predicted forced vital capacity, PEF (%pred) = percentage of predicted for peak expiratory flow, FEV_1_/FVC (%pred) = percent predicted for the ratio of forced expiratory volume in 1 s and forced vital capacity.

**Table 2:** Percentage of mass aerosol deposition across compartments with different flow rates using the heated/humidified HFNC system.

Compartment	10 L/min (*n* = 8)	30 L/min (*n* = 7)	50 L/min (*n* = 8)	*p*-Value
Lung (%)	17.23 ± 6.78	5.71 ± 2.04 *	3.46 ± 1.24 **	<0.001 ^#^
Upper airway (%)	34.48 ± 10.25	42.10 ± 13.92	46.07 ± 8.45	0.213
Stomach (%)	0.37 ± 0.15	1.05 ± 1.12	0.35 ± 0.49	0.116
Nebulizer (%)	13.57 ± 7.42	9.43 ± 6.30	10.30 ± 7.12	0.437
Cannula (%)	8.78 ± 3.63	13.18 ± 3.32 ***	18.40 ± 3.48 **	<0.001
Tubing (%)	21.93 ± 4.90	24.99 ± 9.31	23.41 ± 7.17	0.720
Humidifier (%)	12.21 ± 5.47	16.79 ± 9.68	10.95 ± 1.87	0.201 ^#^
Exp Filter (%)	11.40 ± 3.65	5.68 ± 3.17 *	6.82 ± 4.00	0.014

Data is expressed as percentage (mean ± standard deviation). One-way ANOVA and ^#^ Kruskal–Wallis Test. *p* < 0.05. * 10 L/min × 30 L/min, ** 10 L/min × 50 L/min, *** 30 L/min × 50 L/min.
